# Coronary Artery Disease in Patients with Chronic Kidney Disease: A Clinical Update

**DOI:** 10.2174/1573403X10666140214122234

**Published:** 2013-11

**Authors:** Qiangjun Cai, Venkata K. Mukku, Masood Ahmad

**Affiliations:** 1 Department of Cardiology, McFarland Clinic, Ames, Iowa, USA; 2 Division of Cardiology, University of Texas Medical Branch, Galveston, Texas, USA

**Keywords:** Coronary artery disease, chronic kidney disease, end-stage renal disease, percutaneous coronary intervention.

## Abstract

Chronic kidney disease (CKD) is an independent risk factor for coronary artery disease (CAD). Coronary artery disease is the leading cause of morbidity and mortality in patients with CKD. The outcomes of CAD are poorer in patients with CKD. In addition to traditional risk factors, several uremia-related risk factors such as inflammation, oxidative stress, endothelial dysfunction, coronary artery calcification, hyperhomocysteinemia, and immunosuppressants have been associated with accelerated atherosclerosis. A number of uremia-related biomarkers are identified as predictors of cardiac outcomes in CKD patients. The symptoms of CAD may not be typical in patients with CKD. Both dobutamine stress echocardiography and radionuclide myocardial perfusion imaging have moderate sensitivity and specificity in detecting obstructive CAD in CKD patients. Invasive coronary angiography carries a risk of contrast nephropathy in patients with advanced CKD. It should be reserved for those patients with a high risk for CAD and those who would benefit from revascularization. Guideline-recommended therapies are, in general, underutilized in renal patients. Medical therapy should be considered the initial strategy for clinically stable CAD. The effects of statins in patients with advanced CKD have been neutral despite a lipid-lowering effect. Compared to non-CKD population, percutaneous coronary intervention (PCI) is associated with higher procedure complications, restenosis, and future cardiac events even in the drug-eluting stent era in patients with CKD. Compared with PCI, coronary artery bypass grafting (CABG) reduces repeat revascularizations but is associated with significant perioperative morbidity and mortality. Screening for CAD is an important part of preoperative evaluation for kidney transplant candidates.

## INTRODUCTION

Chronic kidney disease (CKD) is a major health problem worldwide. Approximately 20 million adults in the United States have CKD with or without decreased glomerular filtration rate (GFR). According to the USRDS (United States Renal Data System), 571,414 patients had end-stage renal disease (ESRD) and 172,553 patients had transplanted kidneys in the United States in 2009 [1]. 

Chronic kidney disease is an independent risk factor for the development of coronary artery disease (CAD) [1]. Coronary artery disease is the leading cause of morbidity and mortality in patients with CKD [2]. Atypical presentation of CAD in patients with CKD often leads to delay in diagnosis and treatment [3]. Furthermore, the outcomes of CAD are poorer in patients with CKD than non-CKD counterparts. This review highlights recent advances in the epidemiology, pathogenesis, diagnosis, and management of CAD among CKD patients.

## PREVALENCE OF CAD IN PATIENTS WITH CKD

Patients with even mildly reduced GFR (30-60 ml/min) are at increased risk for obstructive CAD [4]. The risk increases as renal function declines. In a cohort of dialysis patients with or without angina who underwent coronary angiography, 63% patients had significant CAD defined as ≥75% stenosis of a major coronary artery, with an average of 3.3 lesions per patient. Angiographic CAD (≥50% stenosis) was found in 16 of 30 asymptomatic ESRD patients before initiating dialysis [5]. Renal transplant recipients have a lower CAD prevalence of 15%; however, it is still twice that of the general population. 

**Fig. (1):**
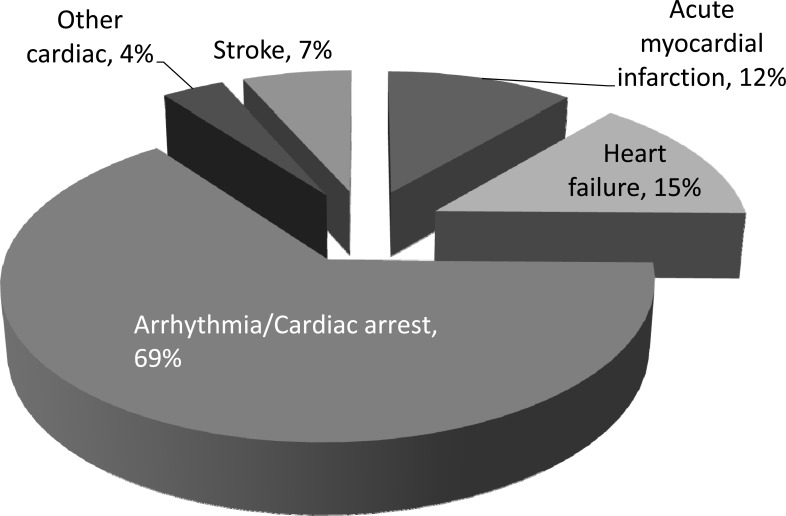
Major causes of cardiovascular death in dialysis patients.

Coronary artery disease contributes to 40% to 50% of deaths among patients who receive dialysis (Fig. **1**) [6]. Approximately 10%-20% of these deaths are due to acute myocardial infarction (AMI) which tends to occur shortly after the initiation of dialysis with 29% within 1 year and 52% within 2 years [7]. Zebrack *et al.* reported a hazard ratio for AMI or death of 2.3 in patients with GFR 30-60 ml/min and 5.1 for GFR <30 ml/min during a 3-year follow up. In this cohort, CKD patients with normal initial angiography also demonstrated increased AMI (5.2% vs 0.7% in non-CKD patients) during follow up, suggesting accelerated progression of CAD. This is corroborated by Gradaus’s study showing that 50% of dialysis patients developed new significant stenosis (≥50%) in a follow up of 30 months. The ability of CKD (GFR<60 ml/min) in predicting future cardiac events, such as myocardial infarction (MI), is at least as good as diabetes, history of MI, obstructive CAD on angiography, and ischemia on stress test [8]. Therefore, CKD is not only an independent risk factor for CAD, but advanced CKD (stages III-V) has also been considered a CAD risk equivalent [9].

## PROGNOSIS OF CAD IN PATIENTS WITH CKD

The impact of CKD on the prognosis of CAD is best illustrated by the survival after AMI. Herzog *et al.* reported a 1-year survival rate of 40.7% in dialysis patients after AMI, while 72% patients died within 2 years [7]. The in-hospital mortality for patients with AMI was 2% in non-CKD, 6% in mild CKD (50 ml/min<GFR≤ 75 ml/min), 14% in moderate CKD (35 ml/min ≤GFR≤ 50 ml/min), 21% in severe CKD (GFR<35 ml/min), and 30% in dialysis patients [10]. The 30-day mortality for ST-elevation MI (STEMI) patients who received thrombolytic therapy was inversely correlated with renal function in a meta-analysis [11]. Patients with mild-to-moderate CKD and non-ST elevation acute coronary syndrome (ACS) had higher 30- and 180-day mortality than non-CKD patients [12]. Patients with diabetic nephropathy have a higher mortality after ACS than patients with other causes of ESRD. In CAD patients, the risk of sudden cardiac death is increased by 11% for every 10 ml/min decline in GFR. The survival after AMI is signiﬁcantly greater in patients who have been transplanted compared to those on the waiting list.

## PATHOGENESIS OF CAD IN PATIENTS WITH CKD

### Traditional Risk Factors 

The prevalence of traditional cardiovascular risk factors such as diabetes, hypertension, and hyperlipidemia is very high in CKD patients. Diabetic nephropathy accounts for 40% of newly diagnosed ESRD. Depending on the cause and severity of CKD, the prevalence of hypertension ranges from 60% to 100%. Dyslipidemia including elevated triglyceride, low-density lipoprotein (LDL), and lipoprotein(a), and decreased high density lipoprotein are typical lipid profiles in dialysis patients. However, the extent and severity of CAD in ESRD is disproportionate to the traditional risk factor profile [13]. This is best exemplified in young ESRD patients with childhood-onset CKD where traditional atherosclerosis risk factors are lacking [1]. Recent research has focused on uremia-related risk factors.

### Inflammation, Oxidative Stress, and Endothelial Dysfunction

Atherosclerosis is a chronic inflammatory disease with increased production of reactive oxygen species involved in atheroma formation. Coronary plaques in ESRD patients are characterized by increased accumulation and activation of macrophages compared with non-renal controls. As renal function deteriorates, plasma levels of pro-inflammatory cytokines (interleukin-6, tumor necrosis factor-α, monocyte chemotactic protein-1) and inflammatory markers (C-reactive protein) increase [14]. In ESRD, a number of dialysis-related and dialysis-unrelated factors (such as infection) may contribute to a state of chronic inflammation. Markedly increased oxidation and impaired anti-oxidation systems are characteristic of ESRD, at least partially due to malnutrition and hypoalbuminemia [15]. Reduced nitric oxide synthesis has an important role in the pathogenesis of endothelial dysfunction and atherosclerosis. Coronary flow reserve, an index of coronary microcirculation and endothelial dysfunction, is significantly lower in CKD patients than the non-CKD group [16]. Furthermore, the use of antioxidants has been shown to improve cardiovascular outcomes in patients with CKD. 

**Fig. (2):**
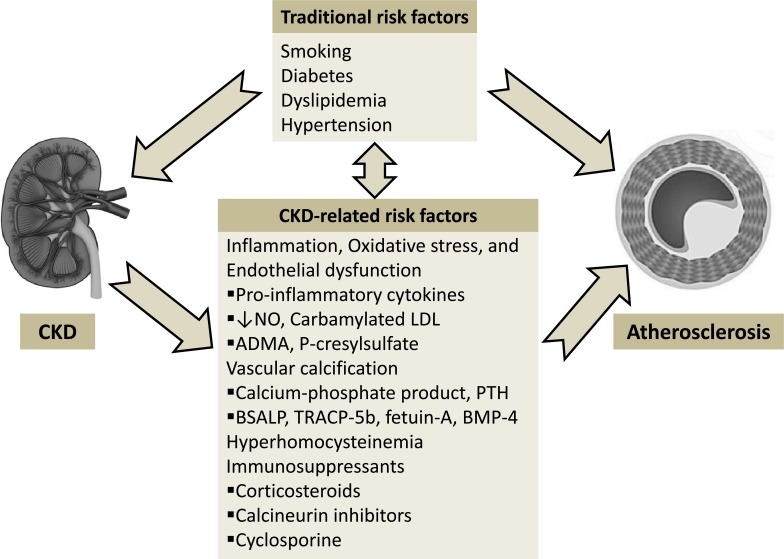
Pathophysiology of progressive atherosclerosis in chronic kidney disease.

Several novel uremia-specific metabolites have been found to be the potential link between CKD and accelerated atherosclerosis (Fig. **2**). Cyanate, a metabolite of urea, modifies LDL to form carbamylated LDL (cLDL). Carbamylated LDL has been recently shown to induce endothelial cell injury, increased expression of cell adhesion molecules, and generation of oxidants. The level of cLDL is elevated in hemodialysis patients and is an independent predictor for the risk of CAD, MI, and death. Asymmetric dimethylarginine (ADMA), an endogenous nitric oxide synthase inhibitor, is elevated in CKD patients and predicts cardiac events [17]. P-cresylsulfate, a uremic factor, has been linked to endothelial dysfunction and major adverse cardiac events among CAD patients [18]. 

### Coronary Artery Calcification

Coronary artery calcium (CAC) score is an independent predictor of cardiac events in both the general population and CKD patients. The cardiovascular mortalities for dialysis patients with a CAC score of 0-105, 110-1067, and >1094 were 3.0%, 22.4%, and 26.9%, respectively [19]. The prevalence of CAC in different stages of CKD varies from 13.9% in stages I and II up to 83% in stages III–V. The prevalence and extent of CAC are increased in patients with ESRD even in young adults [20]. The Dallas Heart Study showed that CAC scores >400 were 8-fold more prevalent in stages III-V CKD compared with patients without CKD. This association is substantially stronger in diabetics [21]. Coronary calcification can be slowed or arrested after renal transplantation. New evidence suggests that CAC is associated with inflammation which is accelerated in CKD [22]. C-reactive protein has been associated with vascular calcification in patients on peritoneal dialysis. By using virtual histology-intravascular ultrasound, Kono *et al.* recently found that the plaque composition of coronary culprit lesions changed from necrotic core-rich to calcium-rich plaques as renal function decreased, suggesting that such coronary culprit composition was associated with stability, particularly in advanced CKD [23].

In dialysis patients, CAC is correlated with a number of uremia factors related to abnormal calcium and phosphate metabolism [20]. The Spokane Heart Study reported that higher levels of serum phosphorus predicted evolution of CAC [24]. Ganesh *et al.* showed a strong relationship between elevated serum phosphate, calcium-phosphate product, parathyroid hormone (PTH), and death from CAD [25]. Markers of bone formation (BSALP, bone-specific alkaline phosphatase) and resorption (TRACP-5b, tartrate-resistant acid phosphatase-5b) can serve as predictors of cardiovascular morbidity and mortality in CKD. Reduced serum levels of calcification inhibitor fetuin-A is associated with increased cardiovascular mortality in dialysis patients. Bone morphogenetic protein-4 (BMP-4), an osteogenic factor, is elevated in patients with both CKD and CAD and positively correlated with the CAC score [26]. 

### Hyperhomocysteinemia

Hyperhomocysteinemia is a potent risk factor for atherosclerosis by inducing oxidative stress and vascular endothelial injury. Abnormal homocysteine metabolism and very high homocysteine levels are consistently found in CKD especially in ESRD patients [27]. However, studies utilizing homocysteine-lowering therapy have failed to show cardiovascular benefit in patients with CKD. 

### Immunosuppressants

Immunosuppressive medications such as corticosteroids, calcineurin inhibitors, and cyclosporine used after renal transplantation have been shown to adversely affect cardiovascular risk factors including hypertension, hyperlipidemia, and hyperglycemia.

## DIAGNOSIS OF CAD IN PATIENTS WITH CKD 

### Symptoms

Symptoms of angina are often misleading in patients with CKD. There is a high prevalence of silent myocardial ischemia owing to diabetic or uremic neuropathy. Angiographically significant CAD was found in 30% to 75% of asymptomatic ESRD patients [28]. On the other hand, the specificity of chest pain for CAD is reduced in CKD. About 25% of ESRD patients with “angina” have no significant stenosis of coronary arteries, probably due to small-vessel disease, microcirculatory dysfunction, or anemia. Dyspnea on exertion is also less specific for angina as it may be secondary to anemia, volume overload, or metabolic acidosis. The sensitivity and specificity of chest symptoms as a predictor of CAD in one study were 51% and 59%, respectively [29]. 

### Electrocardiogram (ECG)

The diagnostic value of baseline ECG is often limited by non-specific changes due to left ventricular hypertrophy and electrolyte disturbances. While exercise ECG testing is helpful in diagnosing CAD, less than half of dialysis patient reach target heart rate secondary to poor exercise tolerance. In a recent study of renal transplant candidates, resting ECG abnormalities (pathological Q wave, left ventricular hypertrophy, ST depression or elevation ≥ 1 mm, T wave inversion or bundle branch block) were strongly predictive of CAD with a sensitivity of 77% but a specificity of only 58%; while exercise ECG had a sensitivity of only 35% [29]. 

### Cardiac Biomarkers

Creatine kinase (CK), CK-MB, and cardiac troponin T may not be useful in the diagnosis of ACS in patients with CKD because of elevated baseline values. Based on different reports, 8%-21% of CKD patients can have abnormal CK and CK-MB, while 10% to 30% of patients have elevated troponin T without evidence of myocardial injury. Cardiac troponin I may be more specific in this population [30].

**Computed Tomography (CT)**

CT is a valuable tool to assess the presence and extent of CAC. Although the prevalence of CAC in renal patients is high, there is no clear correlation between the extent of CAC and the degree of coronary stenosis on invasive angiography [31]. CT coronary angiography is not the optimal test to diagnose CAD in patients with severe CKD due to concerns of contrast-induced renal injury and artifact from extensive coronary calcification. 

### Echocardiography and Radionuclide Stress Imaging

Noninvasive cardiac stress testing has been well studied in patients with CKD, although the sensitivity and specificity are highly variable. Dobutamine stress echocardiography (DSE) in dialysis patients has a sensitivity of 75% to 95%, specificity of 76% to 94%, and accuracy of 90% for the detection of CAD [32]. Sharma *et al.* reported positive and negative predictive values of 86% and 95%, respectively, for DSE to detect significant CAD in renal transplant candidates [33]. A normal DSE identified a very low risk population with a 97% probability of being free of cardiac events or death in ESRD during a 12±6 months follow-up [32]. 

Dipyridamole radionuclide stress testing has a sensitivity of 80% to 86%, specificity of 73% to 79%, and a negative predictive value of 83% in ESRD patients. In patients with suspected CAD, CKD is associated with more extensive ischemia and other high risk features such as transient ischemic dilation on nuclear perfusion testing. The presence of CKD conferred a several-fold higher risk of cardiac death for various strata of perfusion defects [34]. The common causes of a false-negative nuclear perfusion study include single-vessel disease, balanced multivessel disease with global ischemia, and collaterals that prevent the detection of differential flow changes. In addition to ischemia assessment, the presence of reduced coronary flow reserve in patients with moderate to severe renal dysfunction, as assessed by positron emission tomography, is a powerful and independent predictor of cardiac mortality and provides meaningful incremental risk stratification over conventional clinical risk markers [35].

A recent meta-analysis of 12 studies evaluated the prognostic value of DSE and nuclear myocardial perfusion testing in patients with ESRD [36]. Patients with a positive test had a signiﬁcantly greater risk of MI and cardiac death than patients with a negative study. The sensitivities for a future MI or cardiac death with a positive test in this meta-analysis were 70% and 80%, respectively. Both reversible and fixed abnormalities were associated with an increased risk of cardiac death, while the risk of MI was increased only in individuals with reversible defect.

### Invasive Coronary Angiography

The gold standard for the diagnosis of CAD remains coronary angiography. In 126 renal transplant candidates who underwent coronary angiography, significant coronary stenosis (≥70%) on angiography was the single significant predictor of post-transplant cardiac events. The event-free survival was 94% at 48 months in those without and 54% in those with signiﬁcant CAD [37]. However, angiography is an invasive test with a high risk of contrast nephropathy precipitating the need for chronic dialysis in patients with advanced CKD. It should be reserved for those with a high risk for CAD (symptoms of CAD or strongly positive stress test) and those who would benefit from revascularization. In patients with CKD undergoing coronary angiography, iso-osmolar contrast may be better than low-osmolar to reduce contrast-induced nephropathy. 

## MANAGEMENT OF CAD IN PATIENTS WITH CKD 

Management of CAD in patients with CKD can be challenging. Patients with advanced CKD are often excluded from major trials. Therefore, our knowledge in this population is mainly based on extrapolation from the general population, small non-randomized trials, and subgroup analysis. 

**Optimizing medical management:** In general, the management of CAD in CKD patients should be similar to the general population [38, 39]. However, underutilization of aspirin, beta-blockers, angiotensin-converting enzyme inhibitors, glycoprotein IIb/IIIa receptor antagonists, and thrombolytic therapy has been observed in CKD patients due to concerns of bleeding risk, worsening of renal function, and comorbidities. Newsome *et al.* reported that fewer STEMI patients with CKD received thrombolytic therapy, and those with the worst kidney function waited the longest. Interestingly, the odds ratio (OR) for bleeding events was lower in ESRD than in patients with normal kidney function (OR 1.84 vs 2.28, respectively) [40]. De Lima *et al.* reported in a registry that most patients with angiographic CAD on dialysis can be safely managed with medical therapy as the initial strategy [41]. In a post hoc analysis of the COURAGE trial, optimal medical therapy was not inferior to percutaneous coronary intervention (PCI) in stable CAD patients with CKD [42]. In patients with stable CAD, trandolapril was associated with a reduction in total mortality in patients with reduced renal function but not with preserved renal function. In ESPRIT trial, as an adjunct to stent implantation, platelet glycoprotein IIb/IIIa inhibitor therapy on 30-day outcome trended toward a greater magnitude in patients with lower GFR (60 ml/min) compared with those with higher GFR (90 ml/min). These studies suggest that reduced renal function may define a subset of patients more likely to benefit from certain medical therapy. In patients presenting with non-ST elevation MI (NSTEMI) found to have significant CAD on coronary angiography and managed medically, CKD is strongly associated with in-hospital mortality and bleeding [43] . Nonrevascularized patients with CKD have more comorbidities than patients without CKD and less frequently receive guideline-recommended therapies. 

Meta-analyses and post-hoc analyses of studies in patients with mild-to-moderate renal dysfunction have noted benefits with statin therapy. Despite significant reductions in serum LDL levels, the 4-D, AURORA and SHARP trials found no definite clinical benefit with statin therapy in hemodialysis patients [44, 45]. However, the SHARP trial showed a trend towards benefit in atherosclerotic events, and post hoc analyses of AURORA and 4-D showed benefits among diabetic patients and among those with an LDL concentration greater than 145 mg/dL. The most likely explanation for these discordant results is that the pathogenic processes for adverse cardiovascular outcomes among patients with ESRD differ from those with either mild to moderate renal dysfunction or normal kidney function. Therefore, it is not routinely recommended to initiate statin therapy in dialysis patients despite being at high cardiovascular risk.

## PERCUTANEOUS CORONARY INTERVENTION (PCI) 

Patients with CKD are more likely to have three-vessel or left main disease than those without CKD [46]. Lesion complexity increases progressively with decreasing kidney function with GFR a strong predictor of higher SYNTAX (SYNergy between PCI with TAXUS™ and Cardiac Surgery) Score [47]. However, patients with impaired renal function are much less likely than patients with normal renal function to undergo PCI. CKD patients presenting with NSTEMI and managed with PCI receive guideline-recommended therapies less frequently than do patients without CKD. CKD is strongly associated with in-hospital mortality and bleeding in NSTEMI patients undergoing PCI [48]. 

Patients with CKD have substantially elevated risk of adverse clinical outcomes after coronary revascularization compared to non-CKD population [49]. There is a significantly increased risk of cerebrovascular events and bleeding complications in patients with impaired renal function, especially in hemodialysis patients. Recurrent ischemia was observed in 63%, MI in 23%, and death in 13% of dialysis patients 6 months after angioplasty. The 1-year mortality was inversely correlated with GFR with 1.5% in GFR 70-90 ml/min and 19.9% in dialysis patients. Percutaneous coronary intervention in AMI showed a higher 30-day death rate (7.5%) in CKD versus non-CKD patients (0.8%) [50]. Dragu *et al.* reported that CKD patients with STEMI had a lower 30-day mortality with thrombolysis (8.3%) than PCI (37.1%); however, another study revealed that patients with severe CKD and ACS had improved long-term survival when treated with PCI compared to those treated medically. Factors contributing to the unfavorable acute and long-term results of angioplasty in renal patients include complex lesions, diffuse disease, extensive calcification, small vessel diameters, high prevalence of diabetes, and multi-vessel disease [51]. A multivariate analysis showed that CKD was an independent predictor of later additional PCI, suggesting an important role for impaired renal function in the progression of new culprit coronary artery lesions after PCI [52]. Patients with moderate CKD or ESRD undergoing PCI have an approximately threefold increase in the risk of in-hospital mortality compared with patients with preserved renal function [53].

Angioplasty in dialysis patients has high restenosis rate (60% to 81%). Factors associated with restenosis are smaller artery diameter, diffuse disease, and elevated plasma fibrinogen levels. Rubenstein *et al.* found more promising short- and long-term outcomes using stents and debulking devices. Drug-eluting stent (DES) use and intracoronary brachytherapy lowered the rates of restenosis and target lesion revascularization [54]. However, an analysis from the National Heart, Lung, and Blood Institute Dynamic Registry demonstrated that the use of DES was associated with a decreased need for repeat revascularization in the normal-GFR group but not in the low-GFR group. The effect of DES in decreasing repeat revascularization appeared to be attenuated in renal patients [55]. A graded relationship was observed between lower renal function and increased target vessel revascularization and cardiac death after paclitaxel-eluting stent implantation. In diabetic patients with CAD taking maintenance aspirin and clopidogrel therapy, impaired renal function is associated with reduced clopidogrel-induced antiplatelet effects and a greater prevalence of high post-treatment platelet reactivity [56]. The incidence of 1-year definite or probable stent thrombosis was significantly higher in CKD patients compared with patients with normal renal function after DES implantation (1.8% vs 0.6%, P = 0.014) [57]. 

**Fig. (3):**
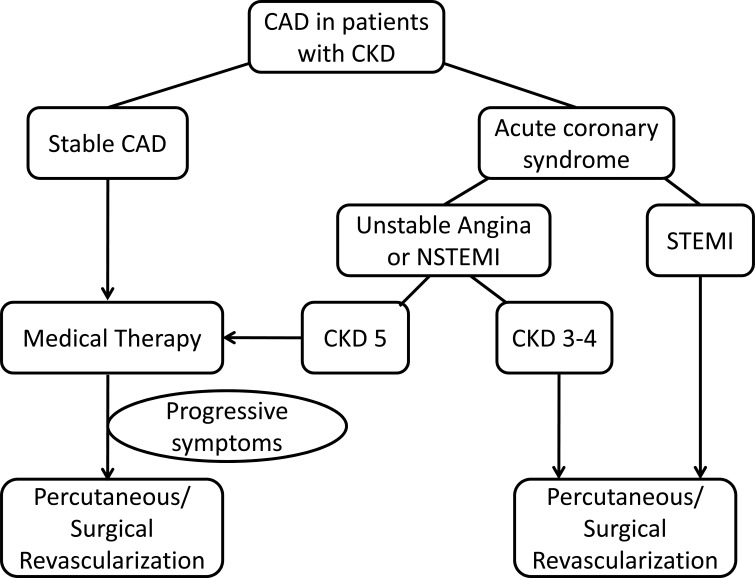
Pathophysiology of progressive atherosclerosis in chronic kidney disease.

**PCI in ACS**: Although there are no randomized trials of reperfusion therapy in CKD, primary PCI, when available, should be the treatment of choice irrespective of CKD severity [38]. In most patients, the cardiovascular risk is greater than the risk of requiring renal replacement therapy. Thus, clinical decisions must be individualized. Among patients with non-ST elevation ACS, an early invasive strategy is recommended in high-risk patients in the general population. A recent meta-analysis suggested similar benefits in CKD 3–4 patients [58]. Although patients with predialysis CKD are less likely to be offered coronary angiography for non-ST elevation ACS, studies suggest a benefit of revascularization with a reduction in 6-month mortality [51]. For ESRD patients or those on dialysis, data are not supportive for PCI. The SWEDEHEART study suggested that an early invasive strategy was harmful for CKD 5 patients (Fig. **3**) [59]. It is likely that competing mortality from other sources would reduce any measured benefit of PCI in ESRD.

**Coronary artery bypass graft (CABG):** Coronary artery bypass surgery is associated with a survival benefit compared to medical management regardless of renal function. Cooper *et al.* reported that operative mortality increased with declining level of GFR (1.3% in stage 2 CKD and >9% in stages 4 and 5 CKD). They found that preoperative GFR was one of the most powerful predictors of operative mortality and morbidity [60]. Hillis *et al.* evaluated the importance of GFR on overall mortality after CABG in non-dialysis dependent patients. After a median follow-up of 2.3 years, GFR was significantly lower in patients who died during follow up compared with survivors. For every 10 ml/min higher GFR, overall mortality was reduced by 20% and 30-day postoperative mortality by 32% [61]. Dialysis patients undergoing CABG have a high perioperative mortality of approximately 7% to 10% which is at least three times higher than in non-uremic patients. In addition, uremic patients have more bleeding and infection complications. Long-term outcome of dialysis patients after CABG is poor with a 5-year mortality estimated at 48% vs 15% in non-CKD patients. A few studies reported CABG outcomes in mild or moderate CKD. In prospective studies, mildly elevated creatine increased the length of hospital stay, the need for postoperative dialysis, and in-hospital mortality. In another analysis of mild to moderate CKD patients, in-hospital CABG mortality was 11% and survival at 10 years was 32%, similar to dialysis patients [62]. In a study of 131 patients, renal transplant recipients had better outcomes after CABG with a perioperative mortality of 3.2%. Decreasing GFR is also associated with an increased risk of saphenous vein graft occlusion [63].

## COMPARISON OF PCI AND CABG

In general, CABG is associated with an increased perioperative morbidity and mortality but better long-term survival and freedom from angina compared with PCI [64]. In CKD patients with multi-vessel CAD, treatment with CABG or PCI with multi-vessel stenting led to similar outcomes of death, MI, or stroke. However, CABG was associated with decreased repeat revascularizations compared to PCI with DES [65]. Coronary bypass surgery is associated with greater mortality reduction than PCI in severe CKD but not in patients with normal or moderate renal failure. A recent analysis using the USRDS data showed that in 5349 renal transplant recipients, patient survival at 2 years after revascularization was similar in patients undergoing coronary stenting (82.5%), percutaneous transluminal coronary angioplasty (PTCA) (82.1%), and CABG using internal mammary grafts (82.7%) [66]. Risk of cardiac death or AMI was lowest in the group treated with internal mammary grafts compared to PTCA. 

## CORONARY ARTERY DISEASE SCREENING IN RENAL TRANSPLANT CANDIDATES 

Renal transplantation is the treatment of choice for patients with ESRD. Cardiovascular events cause 35% to 50% of all deaths after transplantation and CAD is responsible for about 50% perioperative deaths. In face of a limited organ supply, all candidates should be screened for CAD prior to transplantation [67]. Asymptomatic patients with diabetes or multiple CAD risk factors should undergo noninvasive testing, either stress echocardiography or nuclear myocardial perfusion tests. If the stress test is abnormal, coronary angiography is recommended [67]. Transplant candidates with angina, or diabetics with evidence of ischemia, should undergo coronary angiography directly [67]. There is no consensus regarding the optimal noninvasive test modality. This should be determined by the expertise of the individual center [68]. In patients with an initial normal DSE, the cardiac event rate doubles in 40 months while on the transplant waiting list [69]. Therefore, as the waiting time is still prolonged, it is recommended that non-invasive test should be repeated annually in those high risk patients [70]. 

Prophylactic coronary revascularization in the general population failed to show benefit in patient survival prior to non-cardiac surgery. However, in a small randomized controlled trial of 26 diabetic ESRD patients with asymptomatic CAD, the outcome for those who were revascularized was markedly superior to those managed medically, with only 2 of 13 reaching a cardiovascular endpoint in 8.4 months of follow-up compared to 10 of 13 of the patients managed medically [71]. Accordingly, the current guidelines recommend revascularization before renal transplantation in asymptomatic ESRD patients with critical lesions only [67]. However, if the patient has angina or a strongly positive noninvasive test, a team approach including cardiologists and nephrologists should be adopted to determine whether the patient will beneﬁt from intervention.

## CONCLUSIONS

Coronary artery disease is highly prevalent in patients with CKD and carries a poor prognosis. In addition to traditional risk factors, uremia-specific risk factors play an important role in the rapid progression of CAD in this population. Non-invasive stress testing and coronary angiography are often used to diagnose obstructive CAD in renal patients. Chronic kidney disease patients with stable CAD can be initially managed with optimal medical therapy. Selected patients will benefit from PCI and CABG although they are associated with increased procedure risk and poorer outcomes compared to the general population. Patients at risk before renal transplant should be screened for CAD. The optimal pre-transplant CAD screening and management approaches are still to be defined. 

## ABBREVIATIONS

ACS: = acute coronary syndrome
ADMA: = asymmetric dimethylarginine
AMI: = acute myocardial infarction
BMP-4: = bone morphogenetic protein-4
BSALP: = bone-specific alkaline phosphatase
CABG: = coronary artery bypass graft
CAC: = coronary artery calcium
CAD: = coronary artery disease
CK: = creatine kinase
CKD: = chronic kidney disease
cLDL: = carbamylated low-density lipoprotein
CT: = computed tomography
DES: = drug-eluting stent
DSE: = dobutamine stress echocardiography
ECG: = electrocardiogram
ESRD: = end-stage renal disease
GFR: = glomerular filtration rate
LDL: = low-density lipoprotein
MI: = myocardial infarction
NSTEMI: = non ST-elevation myocardial infarction
OR: = odds ratio
PCI: = percutaneous coronary intervention
PTCA: = percutaneous transluminal coronary angioplasty
PTH: = parathyroid hormone
STEMI: = ST-elevation myocardial infarction
SYNTAX: = synergy between PCI with TAXUS™ and cardiac surgery
TRACP-5b: = tartrate-resistant acid phosphatase-5b
